# Data on accessibility of corporate information and business transparency in Russia

**DOI:** 10.1016/j.dib.2018.09.048

**Published:** 2018-09-21

**Authors:** Evgeny A. Kuzmin, Valentina E. Guseva, Alena V. Fomina

**Affiliations:** a Institute of Economics of the Ural Branch of the Russian Academy of Sciences, Ekaterinburg, Russian Federation; b Ural State University of Economics, Ekaterinburg, Russian Federation; c Tyumen Industrial University, Tyumen, Russian Federation; d JSC “Central Research Institute of Economy Management and Information Systems “Electronics”, Moscow, Russian Federation

**Keywords:** Companies, Corporate information, Entry barriers, Stakeholders, Corporate relations, Business transparency

## Abstract

Empirically based, the data description covers business transparency in Russia and availability of corporate information for interested parties. Entry barriers in the form of a fee for hard copies of documents are perceptible as an important indicator of business publicity. The study made it possible to summarize current data on 5070 Russian enterprises in order to estimate document copying cost differentiation according to the developed model. The sample size made it also possible to ensure high levels of data quality and representativeness. Actual limiting average mean error Δp was 3.47% with 99% of study validity. The analysis relied on regional and sectorial data groupings to show a strength of various impact factors. In view of this, correlation coefficients, average and weighted average cost values, and descriptive statistics became secondary indicators. The cost value distributed along an interval scale is a major empirical result of the research. The examination of the obtained data makes it possible to identify an availability level of corporate information for various stakeholders and the general public. This is a part of civil right enforcement in the field of information control and validity check. Conjugated scientific issues include pricing of non-core services of companies, corporate relations and modelling of market behaviours. By making a representative data set, authors make an effort to fill the fact-based gap available in other disciplines and related to business transparency in Russia.

**Specifications table**

**Table:** 

Subject area	*Economics*
More specific subject area	*Companies, economics of corporate relations*
Type of data	*Table, graphs*
How data was acquired	*Collection of publicly available incorporation data on enterprises and their financial statements from SPARK-Interfax information Agency*[1]*and Interfax Corporate Information Disclosure Centre*[2]
Data format	*Estimated, analysed*
Experimental factors	*The data description deals with the distribution of the cost for making hard copies of documents at enterprises if requested by stakeholders according to established rates*
Experimental features	*The experiment rests on the hypothesis that the cost of document copying is a significant indicator of business transparency in Russia*
Data source location	*Institute of Economics of the Ural Branch of the Russian Academy of Sciences (Ekaterinburg, Russian Federation), Ural State University of Economics (Ekaterinburg, Russian Federation)*
Data accessibility	*Data available in this article*

**Value of the data**

● It is possible to use the data for an alternative assessment of business transparency and barriers when accessing corporate information.
● The data make it possible to consider extra factors in research models of regional and sectorial differentiation between Russian companies.
● The data will be useful for associated research on pricing for non-core services of companies and on market behaviour modelling.
● It is possible to use the data for an analysis of a level of a direct interaction of Russian enterprises with stakeholders.
● The data and techniques make it possible for other scholars to replicate and expand the analysis and check results of similar empirical studies.

## 1 Data

The exposure of research data is a set of entries (which authors have put together in compliance with the empirical model) as of June 2018 about Russian companies and costs that stakeholders incur when request hard copies of the corporate documents. Financial indicators have supplemented the corporate information making it possible to correlate and calculate weighted average values for the cost. For the analysis, authors used key financial indicators of enterprises for the previous reporting year (2016) taken from the SPARK-Interfax Information Agency [1]. They include share capital, net assets, return on sales, net profit margin (ROS), gross profit margin, assets, equity, revenue, and net profit/loss. Preliminary estimates of sample coverage by internal classification criteria (region and unstructured sectors on the register of the Interfax Corporate Information Disclosure Centre [2]) had showed strong differentiation and authors made changes to the list of classification criteria. Federal districts were a regional distribution unit. For a presentation of the data exposure, authors substituted unstructured sectors with aggregative sectors according to the Russian Classifier of Economic Activities (OKVED) [3]. Authors have assigned the sectors with poorly presented companies to “others” [sectors], in order to have higher representativeness of estimated levels of corporate information accessibility.

**Fig. 1:**
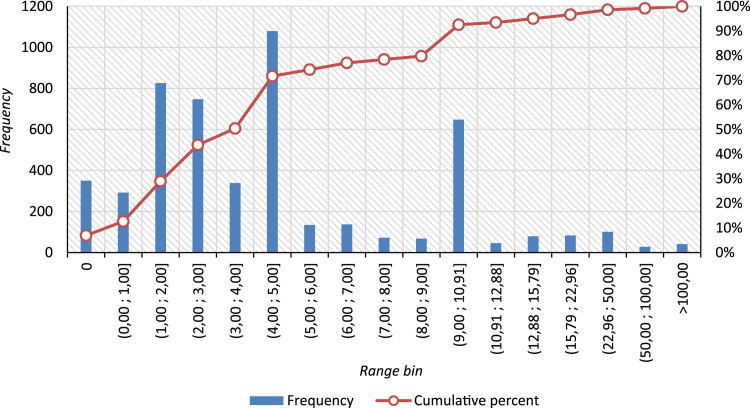
Standard cost distribution for document hard copy making (RUB/page) [as of June, 2018].

**Fig. 2:**
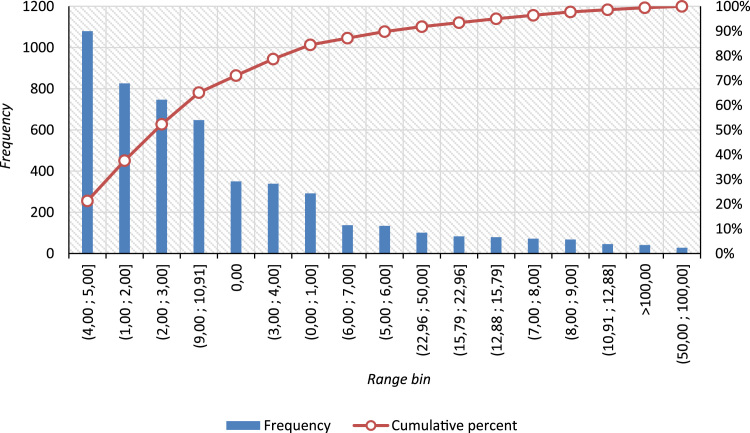
Pareto distribution for document hard copy making (RUB/page) by case frequency [as of June, 2018].

The processed data in the exposure have a form of tables and graphs. Graphs (Fig. 1, Fig. 2) show standard and Pareto distributions of the usual cost for hard copy making for documents if requested by stakeholders for all the companies, regardless of their business and incorporation place.

Tables show calculated values from descriptive statistics on dynamics in values of the cost for corporate document hard copy making, average and weighted average values for compared key indicators (chosen within the empirical study model). Tables also show correlation coefficients for some of the most significant financial indicators. The data set contains detailed calculations by Russian regions (federal districts) (Table 1) and aggregated sectors (Table 2). In compliance with assumed range bins, authors have also presented the integral distribution of usual costs for document hard copy making for both whole Russia, and regions (Table 3) and aggregated sectors (Table 4). Estimated calculations make it possible to complete a system analysis of a free access to corporate information in Russia for subsequent interpretation of trends and recommendation development.

**Table 1:** Descriptive statistics and correlated costs of document hard copy making by Russian regions [as of June 2018].

**Indicators**	**Russia**	**Federal districts of Russia**								
		**SFD**	**CFD**	**UFD**	**SFD**	**NCFD**	**NWFD**	**VFD**	**CFD**	**FEFD**
*I. Descriptive statistics of document hard copy making*										
Average cost, RUB/page	22.96	14.95	20.87	32.53	7.29	49.76	57.96	13.73	5.56	6.62
Standard error	6.27	9.04	7.20	21.82	1.99	43.13	47.46	5.19	1.06	0.59
Median, RUB/page	4.00	2.36	5.00	4.50	3.00	3.00	5.00	4.00	5.00	5.00
Mode, RUB/page	5.00	2.00	5.00	5.00	3.00	2.00	5.00	5.00	5.00	5.00
Standard deviation	446.7	214.9	278.0	469.4	46.7	657.0	1095.8	164.9	4.9	8.5
Sample excess	2176.4	516.9	1124.6	442.8	380.9	230.8	532.2	834.0	3.1	75.3
Sample asymmetry	43.0	22.4	32.1	20.9	18.7	15.2	23.1	27.9	1.7	7.5
Minimum (1), RUB/page	0	0	0	0	0	0	0	0	0	0
Minimum (2), RUB/page	0.17	0.20	0.17	0.22	0.31	0.40	0.25	0.20	2.00	1.00
Maximum, RUB/page	25,300	5,000	10,000	10,000	1,000	10,000	25,300	5000	20	100
Number of observations	5,070	565	1,492	463	551	232	533	1,009	21	204

*II. Average and weighted average values*										
Average number of documents per enterprise for all the time	95	83	99	117	94	61	92	98	27	106
Average number of events per enterprise for all the time	141	112	157	161	138	77	140	140	49	173
Average number of documents and events per enterprise for all the time	237	194	255	278	231	138	232	238	76	279
Average age of enterprises in the sample, yrs.	21.5	22.4	21.5	20.9	21.9	22.8	21.1	21.3	20.7	21.3
WAS on number of enterprises’ documents, RUB/page	15.79	12.19	12.42	8.01	5.75	7.49	73.68	6.70	9.35	6.19
WAS on enterprises’ number of events, RUB/page	10.91	11.34	9.67	12.82	5.69	10.50	23.95	8.67	13.62	7.05
WAS on enterprises’ number of documents and events, RUB/page	12.88	11.70	10.74	10.80	5.71	9.18	43.61	7.86	12.11	6.73
WAS on enterprises’ size of shared capital, RUB/page	5.31	3.46	5.14	6.97	3.86	2.55	8.68	4.79	16.21	6.19
WAS on enterprises’ size of equity, RUB/page	5.15	4.40	4.87	3.72	5.84	83.57	6.19	4.32	−0.04	5.22
WAS on enterprises’ size of net assets, RUB/page	5.15	4.40	4.86	3.72	5.84	83.55	6.18	4.32	0.57	5.23
WAS on enterprises’ revenue, RUB/page	5.64	4.45	4.74	5.11	7.34	35.87	6.53	5.81	2.59	6.24

*III. Cost correlation coefficients*										
Correlation on enterprise׳s number of documents	−0.019	−0.016	−0.030	−0.062	−0.042	−0.064	0.019	−0.062	0.604	−0.069
Correlation on enterprise׳s number of events	−0.016	−0.014	−0.019	−0.031	−0.019	−0.057	−0.023	−0.024	0.672	0.039
Correlation on enterprises’ number of documents and events	−0.018	−0.016	−0.023	−0.045	−0.027	−0.069	−0.013	−0.038	0.659	0.012
Enterprises revenue correlation	−0.003	−0.012	−0.005	−0.009	0.000	−0.003	−0.008	−0.007	−0.407	0.011
Equity correlation	−0.002	−0.008	−0.004	−0.005	−0.004	0.004	−0.008	−0.007	−0.136	−0.016
Shared capital size correlation	−0.002	−0.005	−0.004	−0.010	−0.010	−0.007	−0.004	−0.007	0.677	−0.013
Enterprise age correlation	0.010	0.016	−0.001	0.037	0.043	−0.002	0.030	−0.050	0.535	0.091
Net assets correlation	−0.002	−0.008	−0.005	−0.005	−0.004	0.004	−0.008	−0.007	−0.137	−0.016
Profit (loss) correlation	−0.003	−0.006	−0.004	−0.003	−0.001	−0.001	−0.005	−0.004	−0.187	−0.014

Note: WAS — is weighted average cost; minimum (2) — is minimally non-zero value; SFD — is Southern Federal District; CFD — is Central Federal District; UFD — is Ural Federal District; SFD — is Siberian Federal District; NCFD — is North Caucasian Federal District; NWFD — is Northwestern Federal District; VFD — is Volga Federal District; CFD — is Crimean Federal District; FEFD — is Far Eastern Federal District.

**Table 2:** Descriptive statistics and correlated costs of document hard copy making in Russian enterprises by aggregated sectors [as of June 2018].

**Indicators**	**Russia**	**Types of economic activities (aggregated sectors)**											
		**Section A**	**Section B**	**Section C**	**Section D**	**Section F**	**Section G**	**Section H**	**Section J**	**Section K**	**Section L**	**Section M**	**Other**
*I. Descriptive statistics on the cost of enterprise׳s document hard copy*													
Average cost, RUB/page	22.96	8.88	6.62	21.57	53.75	8.66	34.94	6.00	7.45	6.95	14.71	91.82	10.86
Standard error	6.27	2.71	1.77	7.58	43.65	2.92	29.75	0.53	2.24	0.95	7.22	65.82	2.88
Median, RUB/page	4.00	4.00	3.80	4.50	4.00	4.39	4.32	4.00	2.85	3.50	4.00	5.00	3.50
Mode, RUB/page	5.00	5.00	10.00	5.00	5.00	5.00	5.00	5.00	2.00	0.00	5.00	5.00	5.00
Standard deviation	446.7	61.8	17.9	281.3	662.0	54.2	545.3	11.5	18.0	16.5	190.9	1,306.6	43.3
Sample excess	2,176.4	253.9	86.8	1,154.0	225.3	326.5	335.9	79.8	21.2	158.0	669.0	355.6	83.9
Sample asymmetry	43.0	15.9	9.0	32.8	14.9	17.8	18.3	8.1	4.6	11.3	25.6	18.5	8.6
Minimum (1), RUB/page	0	0	0	0	0	0	0	0	0	0	0	0	0
Maximum (2), RUB/page	0.17	1.00	0.24	0.17	0.50	0.20	0.21	0.40	0.25	0.50	0.31	0.20	0.40
Maximum, RUB/page	25,300	1,000	178.39	10,000	10,000	1,000	10,000	150	100	250	5,000	25,300	500
Number of observations	5,070	521	102	1376	230	346	336	472	65	303	699	394	226

*II. Average and weighted average values*													
Average number of documents per enterprise for all the time	95	62	126	102	151	80	87	87	91	163	82	93	71
Average number of events per enterprise for all the time	141	63	279	156	252	99	113	113	186	386	90	124	75
Average number of documents and events per enterprise for all the time	237	125	405	258	403	179	200	199	277	549	172	216	146
Average age of enterprises in the sample, yrs.	21.5	21.8	21.2	22.9	17.3	21.9	21.8	22.0	19.2	18.6	21.8	21.0	19.9
WAS on a number of enterprises’ documents, RUB/page	15.79	5.08	5.55	12.94	6.77	8.26	5.16	5.80	4.55	6.49	6.60	105.82	8.44
WAS on a number of enterprises’ events, RUB/page	10.91	7.98	4.88	10.43	7.14	6.59	15.29	5.35	3.62	8.45	11.36	36.53	9.00
WAS on a number of enterprises’ documents and events, RUB/page	12.88	6.54	5.09	11.42	7.00	7.34	10.90	5.54	3.93	7.87	9.09	66.21	8.73
WAS on a size of shared capital of enterprises, RUB/page	5.31	5.97	4.85	4.96	3.67	2.68	3.62	5.97	1.50	5.94	4.19	15.63	1.33
WAS cost on a size of equity of enterprises, RUB/page	5.15	5.89	2.96	5.55	6.43	4.00	7.79	5.36	1.23	7.79	5.41	7.15	2.48
WAS on a size of net assets of enterprises, RUB/page	5.15	5.89	2.96	5.63	6.42	4.12	7.79	5.36	1.23	7.01	5.42	7.15	2.48
WAS on enterprises’ revenue, RUB/page	5.64	5.74	2.89	7.37	7.15	5.44	7.63	4.32	1.08	3.47	6.38	10.89	12.81

*III. Cost correlation coefficients*													
Correlation on enterprise׳s number of documents	−0.019	−0.079	−0.099	−0.042	−0.090	−0.009	−0.066	−0.025	−0.190	−0.027	−0.063	0.013	−0.090
Correlation on enterprise׳s number of events	−0.016	−0.013	−0.056	−0.030	−0.048	−0.038	−0.024	−0.040	−0.102	0.061	−0.021	−0.031	−0.040
Correlation on enterprises’ number of documents and events	−0.018	−0.046	−0.065	−0.037	−0.062	−0.030	−0.041	−0.039	−0.123	0.043	−0.044	−0.020	−0.067
Enterprises revenue correlation	−0.003	−0.010	−0.036	−0.010	−0.030	−0.010	−0.007	−0.012	−0.089	−0.071	−0.015	−0.010	0.010
Equity correlation	−0.002	−0.022	−0.038	−0.009	−0.013	−0.018	−0.006	−0.003	−0.087	0.066	−0.004	−0.005	−0.016
Shared capital size correlation	−0.002	−0.009	−0.020	−0.005	−0.011	−0.016	−0.004	0.000	−0.109	−0.011	−0.004	−0.013	−0.018
Enterprise age correlation	0.010	0.010	−0.009	−0.009	0.062	−0.001	0.025	−0.016	−0.062	0.052	−0.065	0.043	0.062
Net assets correlation	−0.002	−0.022	−0.038	−0.009	−0.013	−0.018	−0.006	−0.003	−0.087	0.047	−0.004	−0.005	−0.016
Profit (loss) correlation	−0.003	−0.009	−0.019	−0.005	−0.011	−0.003	−0.005	−0.036	−0.090	0.020	−0.002	−0.005	0.025

Note: WAS — is weighted average cost; minimum (2) — is minimally non-zero value; Section A — Agriculture, forestry, hunting, fishing and fish farming; Section B — Mineral extraction; Section C — Manufacturing; Section D — Supply of power, gas and steam, air conditioning; Section F — Construction; Section G— Wholesale and retail trade, motor vehicle and motorcycle maintenance; Section H — Transportation and storage; Section J — Activities in the field of information and communications; Section K — Financial and insurance business; Section L — Real estate; Section M — Vocational, academic, and technical careers; Others (Section E — Water supply, water disposal, organization of waste collection and disposal, pollution elimination, Section I — Hotels and public catering enterprises, Section N — Administration and related supplementary services, Section O — Public administration and military safety and security and social care, Section P — Education, Section Q — Public health and social support, Section R — Culture, sports, leisure, and entertainment arrangements, Section S — Provision of other types of services).

**Table 3:** Value distribution of the cost for making hard copies of corporate documents by Russian regions [as of June 2018].

**Cost range bin, RUB/page**	**Integral distribution, %**									
	**Russia**	**Federal districts of Russia**								
		**SFD**	**CFD**	**UFD**	**SFD**	**NCFD**	**NWFD**	**VFD**	**CFD**	**FEFD**
0.00*	6.9	8.0	7.0	12.7	9.4	2.6	4.3	4.7	9.5	5.9
(0.00; 1.00]	12.7	14.3	11.9	21.2	16.2	15.1	6.2	10.6	9.5	9.3
(1.00; 2.00]	29.0	49.2	22.1	33.5	36.5	41.8	13.1	29.5	28.6	16.2
(2.00; 3.00]	43.7	64.2	34.0	42.8	61.2	65.1	23.6	45.7	38.1	31.4
(3.00; 4.00]*	50.4	71.3	39.1	48.8	66.1	66.8	38.5	52.8	38.1	37.3
(4.00; 5.00]*	71.7	88.8	62.5	67.6	82.6	86.2	58.7	77.0	76.2	61.3
(5.00; 6.00]	74.3	90.1	65.3	71.5	85.5	87.5	62.3	79.4	76.2	63.2
(6.00; 7.00]	77.0	90.8	67.7	75.2	88.4	88.8	65.9	82.7	76.2	68.1
(7.00; 8.00]	78.4	91.9	68.9	76.7	88.9	88.8	67.7	84.7	76.2	71.6
(8.00; 9.00]	79.8	92.2	69.9	84.2	89.3	88.8	68.9	85.2	81.0	72.5
(9.00; 10.91]*	92.5	98.6	89.7	91.6	95.5	96.1	88.4	93.8	90.5	92.2
(10.91; 12.88]*	93.5	98.6	91.3	92.9	96.0	97.0	88.9	94.3	90.5	93.6
(12.88; 15.79]*	95.0	98.8	93.4	95.0	96.9	97.0	91.6	95.3	95.2	96.6
(15.79; 22.96]*	96.6	98.9	95.6	96.8	98.0	97.4	94.2	96.7	100.0	98.5
(22.96; 50.00]	98.6	99.1	98.5	98.7	98.7	98.3	97.7	98.9	100.0	99.5
(50.00; 100.00]	99.2	99.3	99.1	99.1	99.3	99.1	99.1	99.2	100.0	100.0
(100.00; ∝)	100.0	100.0	100.0	100.0	100.0	100.0	100.0	100.0	100.0	100.0

Note: 0.00 — means that documents are provided without a fee (free of charge); 4.00 — is a cost median in Russia; 5.00 — is a cost mode in Russia; 10.91 — is a weighted average cost by a number of events of Russian companies; 12.88 – is a weighted average cost by a number of documents and events of Russian companies; 15.79 — is a weighted average cost by a number of documents of Russian companies; 22.96 — is an average cost in Russia; SFD — is Southern Federal District; CFD — is Central Federal District; UFD — is Ural Federal District; SFD — is Siberian Federal District; NCFD — is North Caucasian Federal District; NWFD — is North-western Federal District; VFD – is Volga Federal District; CFD — is Crimean Federal District; FEFD — is Far Eastern Federal District.

**Table 4:** Distribution of cost for making hard copies of documents across Russian companies by aggregated sectors [as of June 2018].

**Cost range bin, RUB/page**	**Integral distribution, %**												
	**Russia**	**Types of economic activity (aggregated sectors)**											
		**Section A**	**Section B**	**Section C**	**Section D**	**Section F**	**Section G**	**Section H**	**Section J**	**Section K**	**Section L**	**Section M**	**Other**
0.00*	6.9	3.84	7.84	5.67	10.00	7.51	8.04	5.08	12.31	15.51	6.58	6.35	7.96
(0.00; 1.00]	12.7	7.87	14.71	11.56	14.35	13.58	13.69	10.59	23.08	19.14	13.02	13.71	14.60
(1.00; 2.00]	29.0	28.41	32.35	25.44	31.74	30.64	30.36	29.45	49.23	30.36	29.90	26.65	34.96
(2.00; 3.00]	43.7	47.41	48.04	41.57	43.04	45.95	43.45	45.13	60.00	37.62	45.64	37.31	49.12
(3.00; 4.00]*	50.4	51.82	51.96	48.33	50.87	49.71	49.70	51.48	60.00	55.45	51.22	44.67	55.75
(4.00; 5.00]*	71.7	77.74	69.61	69.11	67.39	73.41	73.81	76.48	80.00	67.99	74.25	60.91	75.66
(5.00; 6.00]	74.3	79.08	73.53	72.17	72.17	75.14	77.38	78.60	80.00	72.61	75.54	64.47	77.88
(6.00; 7.00]	77.0	81.77	76.47	75.44	78.26	77.46	79.17	80.30	81.54	73.93	78.25	67.01	80.09
(7.00; 8.00]	78.4	82.53	78.43	76.74	81.74	78.32	80.95	81.57	83.08	76.57	79.11	68.78	81.42
(8.00; 9.00]	79.8	82.53	80.39	77.76	83.04	79.77	81.25	82.42	83.08	78.55	79.69	76.14	81.42
(9.00; 10.91]*	92.5	95.20	96.08	90.63	91.74	93.35	93.45	94.70	92.31	89.11	93.56	91.12	94.25
(10.91; 12.88]*	93.5	96.35	96.08	91.28	93.48	94.51	94.64	95.76	93.85	89.77	94.28	92.64	94.25
(12.88; 15.79]*	95.0	97.50	97.06	93.90	95.65	95.66	95.54	95.97	93.85	91.42	95.71	94.42	94.69
(15.79; 22.96]*	96.6	98.27	97.06	95.64	96.96	97.40	98.21	97.25	93.85	95.05	97.42	96.45	94.69
(22.96; 50.00]	98.6	99.62	99.02	98.11	97.83	99.13	99.40	99.15	96.92	99.34	98.71	97.97	97.79
(50.00; 100.00]	99.2	99.62	99.02	98.69	98.70	99.71	99.70	99.79	100.00	99.67	99.43	98.73	98.23
(100.00; ∝)	100.0	100.00	100.00	100.00	100.00	100.00	100.00	100.00	100.00	100.00	100.00	100.00	100.00

Note: 0.00 — means that documents are provided without a fee (free of charge); 4.00 — is a cost median in Russia; 5.00 — is a cost mode in Russia; 10.91 — is a weighted average cost by a number of events of Russian companies; 12.88 — is a weighted average cost by a number of documents and events of Russian companies; 15.79 — is a weighted average cost by a number of documents of Russian companies; 22.96 — is an average cost in Russia; Section A — Agriculture, forestry, hunting, fishing and fish farming; Section B – Mineral extraction; Section C — Manufacturing; Section D – Supply of power, gas and steam, air conditioning; Section F — Construction; Section G — Wholesale and retail trade, motor vehicle and motorcycle maintenance; Section H — Transportation and storage; Section J — Activities in the field of information and communications; Section K — Financial and insurance business; Section L — Real estate; Section M — Vocational, academic, and technical careers; Others (Section E — Water supply, water disposal, organization of waste collection and disposal, pollution elimination, Section I — Hotels and public catering enterprises, Section N — Administration and related supplementary services, Section O — Public administration and military safety and security and social care, Section P – Education, Section Q — Public health and social support, Section R — Culture, sports, leisure, and entertainment arrangements, Section S — Provision of other types of services).

## 2 Experimental design, materials, and methods

### 2.1 Study area

The empirical study was organised from the entire assembly, i.e. enterprises that are on the register of the agency accredited by the Bank of Russia for corporate information disclosure. The Interfax Corporate Information Disclosure Centre [2] was a chosen source of raw data. The register included public and privately held companies in the Russian market of securities: joint-stock companies (widely), limited liability companies (narrowly) and state-owned corporations (narrowly). The register included both active companies, and the enterprises that had already ceased their operations. Sampling included several criteria and stages.

Decisions on a composition of the entire assembly were as of the data collection time. As of June 2018, the registry contained 36,798 non-repeated entries on enterprises with various degrees of activism. Herewith, making the entire assembly, authors only reviewed enterprises incorporated in Russia. Authors did not take into account the overseas enterprises that had disclosed corporate information in the Russian market of securities. This information on the distribution by Russian regions and sectors served as a basis for the sample quality assessment at sampling stages. The data for a final analysis were an exposure of the effort in compliance with the empirical model.

### 2.2 Sample

To assess a quality of samples, we calculated a limiting error for the observations made at various sampling stages that had preceded the acquisition of the research data exposure. We calculated the limiting error for confidence levels of 90%, 95%, and 99%. In total, sampling included three stages according to the empirical model and resulted in the sample size of 9366, 6555, and 5070 companies respectively. At stages of the empirical model, the coverage was not less than 13.78% as compared to the entire assembly from the registry of companies (excluding the criteria of activism) with samples. Such a coverage allowed low values of the actual limiting error (Table 5), as well as average, minimum, and maximum limiting error among internal classification criteria in the sample (Table 6). As classification criteria, we had chosen regions of Russia (subjects and federal districts) as an incorporation place, as well as unstructured sectors (according to the scale of the Interfax Corporate Information Disclosure Centre [2]). In 99% of cases, the low limiting error of 2.55–3.47% for the confidence P level points out to representativeness of the sample and the empirical study in progress.

**Table 5:** Sample quality and data exposure.

**Sampling stage**	**Entire assembly (register of companies)**	**Sample size (companies)**	**Coverage, %**	**Share of factor criterion**	**Actual limiting error (∆p), %**		
					***P* = 90%**	***P* = 95%**	***P* = 99%**
1	36,798	9,366	25.45	0.5	1.63	1.94	2.55
2	36,798	6,555	17.81	0.5	1.95	2.32	3.05
3	36,798	5,070	13.78	0.5	2.22	2.64	3.47

Note: *P* – is a reliability/accuracy, and ∆*p* – is a limiting error.

**Table 6:** Data exposure quality by internal classification criteria.

**Indicator**	**Share of factor criterion**	**Limiting error (∆p), %**		
		***P* = 90%**	***P* = 95%**	***P* = 99%**
*I. Internal classification criteria - by region (subject) of Russia [3*rd stage]*				
Average limiting error	0.5	2.38	2.84	3.73
Minimum limiting error	0.5	1.38	1.65	2.16
Maximum limiting error	0.5	5.27	6.27	8.25

*II. Internal classification criteria – by unstructured industry [3*rd stage]**				
Average limiting error	0.5	2.43	2.90	3.81
Minimum limiting error	0.5	1.40	1.66	2.19
Maximum limiting error	0.5	4.92	5.87	7.71

*III. Internal classification criteria – by region (federal district) of Russia [3*rd stage]***				
Average limiting error	0.5	2.21	2.63	3.46
Minimum limiting error	0.5	2.01	2.39	3.14
Maximum limiting error	0.5	2.43	2.90	3.81

Note: *P* – is a reliability/accuracy, ∆*p* – is a limiting error;

* Is 85 subjects of the Russian Federation;

** They used unstructured sectors according to the registry of companies of the Interfax Corporate Information Disclosure Centre [2] (banks; pulp, paper and woodworking industry; investment companies; light industry; mechanic engineering; research institutions; oil and gas production and refining; food industry; production of construction materials; communications; agriculture; insurance companies; construction; fuel industry; trade; transportation; services; chemical industry; holdings and property management companies; ferrous and non-ferrous industry; power sector; other);

*** Stands for nine federal district of the Russian Federation.

As for criteria of internal classification, in the data exposure, companies’ presentation is non-homogeneous. The value range spread of the specific share of companies in unstructured sectors is 31.5 percentage points, from 0.2% (insurance companies) to 31.7% (other companies). There are similar values obvious for regions (federal districts) of Russia, among which, the Central Federal District has the highest representation with a specific share of 29.4% of companies in the exposure, while the Crimean Federal District is the least representative one with its 0.4%. There is a shift in the structure of companies in the sample at the third stage of sampling. This makes it difficult to have a cluster analysis by criteria of internal classification. This limitation does not apply to the general analysis of distribution.

### 2.3 Empirical models

The author׳s research approach includes a distribution analysis of cost for hard copying of corporate documents for stakeholders. Our goal is the identification of barriers that are in place when we offline access such the information and tracing trends towards changes in the development of corporate ethical obligations and business practices in Russia in terms of business transparency. The empirical model assumes consecutive sampling of data that meet the given parameters.

At *the first stage*, we collected initial data on companies from the registry of the Interfax Corporate Information Disclosure Centre [2] with a technical restriction of a recall ratio in a pair of classification criteria “region – industry” with no more than 400 entries. An additional condition for sampling is an active status of a company implying that in January-June, 2018, it posted/published at least one document or event. Based on these criteria, the first sample included 9,366 companies in total.

*The second stage* of sampling focuses on stated cost for making hard (printed) copies of requested documents, as well as banking details for payments. This data unit is a core in the empirical research. Enterprises that had not submitted such a statement were out of the sample. Statements had not been structured or in any way standardized, so their processing was manual with a random check (which might lead to a slightly higher error percent in estimates). Based on sampling results at the stage, the completed intermediate sample included 6,555 companies.

The final *third stage* of sampling presents the research data exposure. The sampling principle relied on incomplete statements. The sample did not include the enterprises that had not clearly referred to costs for document hard copy making. We mean statements that only contain banking details, links to company׳s rates posted on corporate sites or other resources except for a data collection service, or links to rates targeted at deleted documents and pages. The sampling resulted in a database of 5070 companies, for which scholars later held a distribution analysis.

The data exposure has a number of research limitations and assumptions. This is mainly due to pricing for making hard copies of documents. This very criterion is the basis of a comparison and analysis. In our research, the cost is for a page of a document in the national currency. A simulated case has the following description:

a) Consumer is an individual, not affiliated with a company and not a shareholder,
b) Requested document is one-paged,
c) Copy is in A4 format, one sided, black and white, with no duplication,
d) Rates are for copying within 7 days of a request day.
  Note that Russian legislation provides for shorter timeframes for provision of documents to shareholders about general meetings of company׳s participants (up to 5 days). At the same time, it allows extending the standard period for 20 days if there are more than 10 requested documents or their volume is over 200 pages. At its discretion, an enterprise might make a limited number of documents and their volume for delivery higher in an extended period of time, but must fix it with the Charter or other in-house record of a joint-stock company),
e) Due to specifics of the Russian tax legislation, the cost for document copying includes VAT if a company׳s statement contains a proviso saying that there is a need in its payment. In all the other cases, the cost is shown *as is*,
f) Companies׳ statements on fees for document copying were published on different days, but we consider them valid and up-to-date (this assumption may lead to a slightly higher error percentage in estimates if companies have not submitted their updated statements to accredited institution “Interfax Centre for Corporate Information Disclosure” [2]),
g) In the research, authors have ignored the fact that in case of approved corporate rates in place for hard copying, in some cases hard copies are free of charge. This might depend on a subjective managerial decision, when a volume of requested documents is very limited and an aggregate value (to be charged) is insignificant.
